# A robust and efficient AI assistant for breast tumor segmentation from DCE-MRI via a spatial-temporal framework

**DOI:** 10.1016/j.patter.2023.100826

**Published:** 2023-08-16

**Authors:** Jiadong Zhang, Zhiming Cui, Zhenwei Shi, Yingjia Jiang, Zhiliang Zhang, Xiaoting Dai, Zhenlu Yang, Yuning Gu, Lei Zhou, Chu Han, Xiaomei Huang, Chenglu Ke, Suyun Li, Zeyan Xu, Fei Gao, Luping Zhou, Rongpin Wang, Jun Liu, Jiayin Zhang, Zhongxiang Ding, Kun Sun, Zhenhui Li, Zaiyi Liu, Dinggang Shen

**Affiliations:** 1 School of Biomedical Engineering, ShanghaiTech University, Shanghai 201210, China; 2 Department of Radiology, Guangdong Provincial People’s Hospital, Guangdong 510080, China; 3 Department of Radiology, The Second Xiangya Hospital, Central South University, Hunan 410011, China; 4 School of Medical Imaging, Hangzhou Medical College, Zhejiang 310059, China; 5 Department of Radiology, Shanghai General Hospital, Shanghai Jiao Tong University School of Medicine, Shanghai 200080, China; 6 Department of Radiology, Guizhou Provincial People’s Hospital, Guizhou 550002, China; 7 School of Health Science and Engineering, University of Shanghai for Science and Technology, Shanghai 200093, China; 8 Department of Medical Imaging, Nanfang Hospital, Southern Medical University, Guangzhou 510515, China; 9 School of Electrical and Information Engineering, The University of Sydney, Sydney, NSW 2006, Australia; 10 Department of Radiology, Key Laboratory of Clinical Cancer Pharmacology and Toxicology Research of Zhejiang Province, Hangzhou 310003, China; 11 Ruijin Hospital, Shanghai Jiao Tong University School of Medicine, Shanghai 200025, China; 12 Department of Radiology, The Third Affiliated Hospital of Kunming Medical University, Kunming 650118, China; 13 Shanghai United Imaging Intelligence Co., Ltd., Shanghai 200230, China; 14 Shanghai Clinical Research and Trial Center, Shanghai 200052, China

## Abstract

Dynamic contrast-enhanced magnetic resonance imaging (DCE-MRI) allows screening, follow up, and diagnosis for breast tumor with high sensitivity. Accurate tumor segmentation from DCE-MRI can provide crucial information of tumor location and shape, which significantly influences the downstream clinical decisions. In this paper, we aim to develop an artificial intelligence (AI) assistant to automatically segment breast tumors by capturing dynamic changes in multi-phase DCE-MRI with a spatial-temporal framework. The main advantages of our AI assistant include (1) robustness, i.e., our model can handle MR data with different phase numbers and imaging intervals, as demonstrated on a large-scale dataset from seven medical centers, and (2) efficiency, i.e., our AI assistant significantly reduces the time required for manual annotation by a factor of 20, while maintaining accuracy comparable to that of physicians. More importantly, as the fundamental step to build an AI-assisted breast cancer diagnosis system, our AI assistant will promote the application of AI in more clinical diagnostic practices regarding breast cancer.

## Highlights

• A robust and efficient AI assistant is developed for breast cancer segmentation
• A large set of DCE-MRI data from seven medical centers is used to train our AI assistant
• A specifically designed spatial-temporal transformer is used to capture dynamics
• Our AI assistant has potential for building an automated breast cancer diagnosis system

## The bigger picture

Breast cancer is the most common cancer affecting women worldwide. Early detection and diagnosis are crucial for better treatment outcomes and improved survival rates. However, high incidence of breast cancer puts a significant burden on clinicians, while training experienced breast imaging clinicians is time-consuming. This raises the need for developing a robust AI assistant for assisting clinicians for breast cancer diagnosis. To this end, we collected a very large set of breast DCE-MRI data from seven medical centers for developing such a breast AI assistant, which can also be used as a foundational step for building an automated breast cancer diagnosis system to facilitate future smart medicine.

## Graphical abstract

**Figure:**
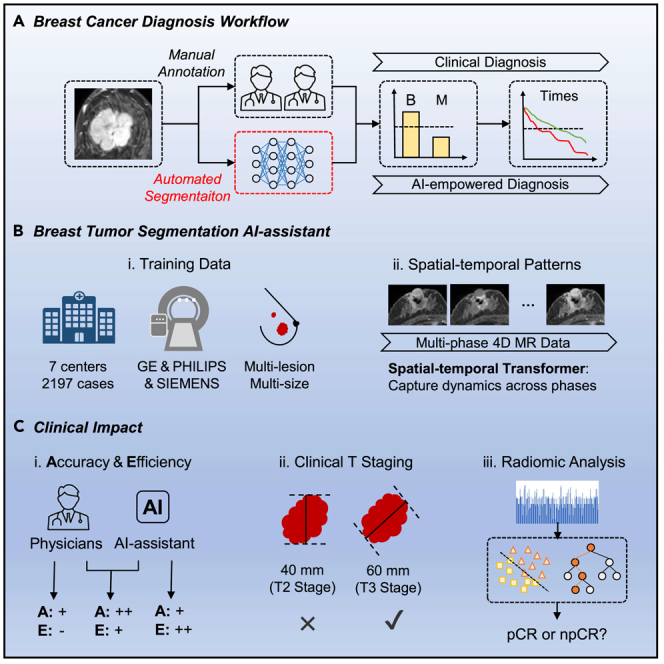

## Teaser

Clinical breast cancer diagnosis can be time-consuming and laborious. In this study, we have developed an AI assistant to automatically segment breast tumors from DCE-MRI data, trained on a very large set of data with a specially designed spatiotemporal transformer. Experimental results show that our AI assistant can produce more accurate results even than clinicians while also using significantly less time. This indicates the potential of our AI assistant in real clinical applications.

## Introduction

Cancer is the second-leading cause of death worldwide.^1^ Among them, breast cancer is the most common malignant neoplasm appearing in women and is becoming more common in the younger population.^2^ Recent studies show that early detection of malignancy with timely clinical intervention will greatly reduce the death risk of patients suffering from breast cancer.^3^^,^^4^ Nowadays, breast X-ray (mammography),^5^^,^^6^ ultrasound,^7^^,^^8^ and magnetic resonance imaging (MRI)^9^^,^^10^^,^^11^ are widely used for screening, localization, and diagnosis of breast cancer. Among these imaging modalities, dynamic contrast-enhanced magnetic resonance imaging (DCE-MRI) has the highest sensitivity to breast tumors and is often used for screening purposes in high-risk populations.^12^^,^^13^ A standard breast DCE-MRI study collects a respective sequence of T1-weighted MR images at multiple phases before, during, and after intravenous administration of the contrast agent.^14^ The tumor-related leaky vasculature leads to fast accumulation and washout of the contrast agent in the tumorous tissue, creating a sharp contrast between soft tissue and tumors.^13^ By comparing signal variation across different image phases, physicians can delineate tumor shape and analyze tumor characteristics for the subsequent examination or treatment plan.

With the development of artificial intelligence (AI) in the medical field, personalized medicine, automated diagnosis, and treatment have become popular topics in recent years,^15^^,^^16^^,^^17^^,^^18^ including AI-assisted breast cancer diagnosis and treatment.^19^^,^^20^^,^^21^^,^^22^^,^^23^ For example, several works try to extract radiomics or deep learning features in breast DCE-MRI data to predict pathological response after neoadjuvant therapies,^24^^,^^25^ and they achieve more than 70% accuracy.^25^ However, accurate diagnosis or treatment prediction relies on accurate tumor annotation, which is tedious and time-consuming even for experienced radiologists.^25^^,^^26^^,^^27^^,^^28^ Semi-automated annotation can significantly compromise the benefit of AI in clinical practice. Hence, building a robust breast tumor segmentation model is a fundamental and crucial step to promote the development of intelligent systems for breast cancer diagnosis.

In the early stage, manually crafted filters or thresholds are used to extract enhanced tissues or contours from DCE-MRI.^29^^,^^30^ These methods greatly rely on the intensity values and ignore semantic information, leading to over-segmentation in background parenchymal enhancement region and under-segmentation in dense breast. Recently, the performance of DCE-MRI-based breast tumor segmentation has been significantly enhanced by the deep learning models, especially the well-known U-Net model,^31^^,^^32^^,^^33^^,^^34^ because these models can automatically learn better segmentation criteria from training data. Particularly, to further distinguish the tumor region from other tissue enhancements, some works exploit tumor shape priors^35^ or design the tumor-sensitive module^36^ to boost segmentation performance. However, most methods only use images from two phases (i.e., one phase without enhancement and the other phase with the strongest tissue enhancement), ignoring dynamic changes across different phases, which greatly limits the capability of the model in capturing the essential characteristics of tumor (e.g., the fast accumulation and washout of contrast agent). Although there exist several works^37^^,^^38^ trying to exploit multi-phase information to assist tumor segmentation, the dynamics and temporal relationships among different phases are not well explored.

Our study aims to develop a robust AI assistant for breast tumor segmentation using DCE-MRI data, by leveraging spatial-temporal relationships across multiple phases. Specifically, we propose a spatial-temporal framework to capture MR signal dynamics for superior performance. Moreover, we introduce a whole-breast segmentation model to localize breast region for ensuring the segmentation model focuses solely on the breast region. To evaluate both accuracy and robustness of our approach, we have collected a large dataset of 13,167 MR volumes from 2,197 cases across seven medical centers. Our extensive studies demonstrate the clinical impact of our AI assistant, which outperforms experienced physicians in clinical diagnosis and treatment design. Our AI assistant has the potential to be a valuable tool for clinical practice, as it significantly improves segmentation efficiency and accuracy.

## Results

### Patients and datasets

In this study, we collect a large set of DCE-MRI data from seven medical centers in China to build a robust and efficient breast AI assistant for tumor segmentation. Specifically, 13,167 DCE-MRI volumes from 2,197 cases of 2,190 patients are used in this study. To our best knowledge, it is the largest breast MRI dataset used for tumor segmentation study so far. The participant centers include Guangdong Provincial People’s Hospital (GD-hospital), Yunnan Cancer Hospital (YN-hospital), Hangzhou First People’s Hospital (HZ-hospital), Shanghai General Hospital (SH-hospital), The Second Xiangya Hospital (XY-hospital), Guizhou Provincial People’s Hospital (GZ-hospital), and Ruijin Hospital (RJ-hospital). More detailed participant information, imaging protocols, and breast region characteristics of each center are listed in Table 1. To be mentioned, most centers collect DCE-MRI data with six phases (one pre-contrast image + five post-contrast images) or eight phases (one pre-contrast image + seven post-contrast images), while some cases such as from RJ-hospital have only two or four phases due to accidental file loss, which is obviously not common in clinical applications. Also, diagnosis information (i.e., BIRADS category or molecular subtype) is unavailable for some centers or patients due to unauthorized diagnosis reports and missing key examinations.

**Table 1:** Description of DCE-MRI from each center

COHORT	GD-hospital	YN-hospital	HZ-hospital	SH-hospital	XY-hospital	GZ-hospital	RJ-hospital
**Participant information**							

Patient number	638	473	273	182	171	48	405
DCE-MRI case number	642	475	274	182	171	48	405
DCE-MRI volumes	3,852	3,688	1,644	1,121	1,074	328	1,460
Examination time	2016–2020	2012–2021	2020–2022	2019–2022	2015–2020	2020–2021	2016–2018
Age (years)	50 (21, 79)	47 (24, 77)	48 (22, 85)	53 (24, 87)	51 (29, 79)	45 (27, 86)	–

**Imaging protocols**							

Phase interval time	1 min	1–2 min	1 min	1 min	1 min	1 min	45 s
Phase number	6	6, 8	6	6, 7, 8	6, 8	6, 8	2, 4, 5, 6, 8
Inter-slice resolution (mm)	0.44–0.98	0.33–0.70	0.75–1.17	0.66–0.93	0.50–1.10	0.63–1.43	0.70–1.00
Slice thickness (mm)	0.5–1.0	0.8–1.7	1.2	1.6	1.0–1.5	0.9–1.2	1.5
Manufacturer	Philips	Siemens, GE	Siemens	Siemens, GE	Siemens	Siemens	Siemens

**Breast region characteristics**							

Breast characteristic (normal/ single/with implant)	640/2/0	472/2/1	269/4/1	178/3/1	166/3/2	47/1/0	399/6/0
Tumor size (S/M/L)	86/369/187	144/235/96	88/87/99	87/56/39	59/49/63	712/20/16	97/206/102
BIRADS category ${}^{2}$ (3/4/5/6)	3/185/89/112	1/23/446/5	75/105/13/81	13/68/97/4	12/36/84/39	0/3/38/7	–
Molecular subtype ${}^{2}$ (luminal A/ luminal B/Her2+/TN)	82/219/36/34	65/304/62/44	–	17/50/18/13	–	9/25/5/3	–

Data description includes participant information, imaging protocols, and breast region characteristic. Dash means unavailable information.Not all the patients have accurate BIRADS category or molecular subtype.

The tumors on the DCE-MRI data are manually annotated and checked by two senior radiologists in each center. Then radiologists from all centers jointly annotate breast tumors. We also invite two experienced raters to label the whole breasts in DCE-MRI from GD-hospital and YN-hospital, which are further checked by radiologists. The BIRADS category is collected from the diagnosis report at examination time. Some patients undergo pathological biopsies to get receptor expression (e.g., estrogen receptor, progesterone receptor, and human epidermal growth factor receptor) and proliferation index (Ki-67) for accurate categorization of molecular subtypes according to the internationally recognized criteria.

**Figure 1:**
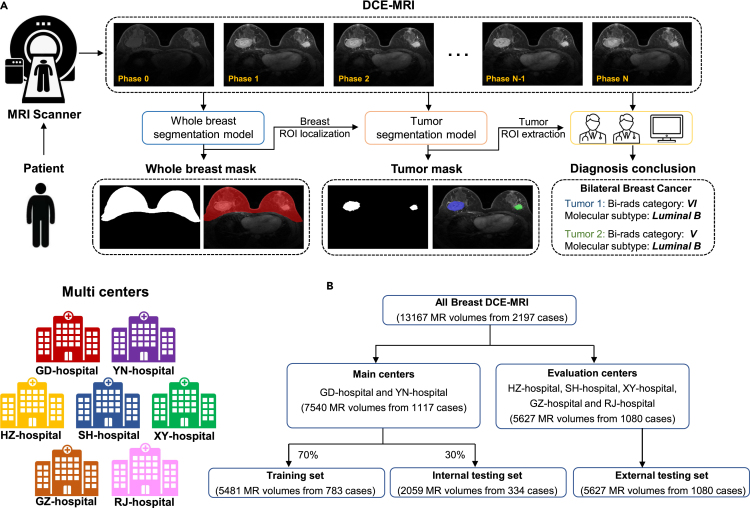
Overview of breast cancer diagnosis process and data information (A) Breast cancer diagnosis process. Multi-phase DCE-MRI data are collected from MRI scanner and then used as inputs to our segmentation models for delineating the whole breast and tumor, which can be further used to guide diagnosis by physician or AI system. (B) Data partition in experiments. Seven centers are included, and we randomly divide them into the training set, internal testing set, and external testing set.

To build a robust and efficient breast AI assistant system for tumor segmentation, we use data from two main centers (GD- and YN-hospital) as the internal set and the rest of the five centers as the external set in our experiments. Then we randomly choose 70% of the data from the internal set to train all the models and the rest of the data (30%) as the internal testing set to evaluate the models’ performance. All cases from the external set are further used to demonstrate the robustness and generalizability of our AI assistant. The detailed data partition is shown in Figure 1B.

In these experiments, three typical segmentation metrics are used to evaluate segmentation performance, including DICE similarity coefficient (DSC, 0%–100%), Sensitivity (SEN, 0%–100%), and Hausdorff distance (HD, mm). DSC measures the similarity between ground truth and the prediction mask. SEN evaluates the under-segmentation ratio. HD calculates the distance between two point sets from ground truth and predicted surfaces. Higher DSC and SEN scores indicate better segmentation performance, while a lower HD score indicates closer boundaries with better results.

### Whole-breast segmentation performance

In clinical practice, physicians only focus on the breast region to localize breast tumors. According to the physician’s diagnosis process, we design a whole-breast segmentation model as the first step in our breast AI assistant to facilitate the downstream tumor segmentation and diagnosis in 3-fold. First, the whole-breast mask can guide accurate patch sampling in the training stage of the tumor segmentation model. Second, it helps our AI assistant to automatically distinguish normal and abnormal breasts. Third, some over-segmentation cases in the non-breast regions, e.g., enhanced tissues in the heart region, can be removed effectively. Therefore, whole-breast segmentation is a crucial first step to significantly improve tumor segmentation performance.

**Figure 2:**
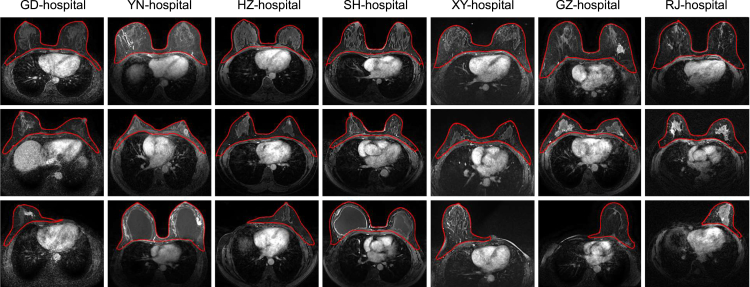
Visualization of whole-breast segmentation results from seven centers Each center (column) is with three typical cases such as standard-sized breasts (top), small-sized breasts (middle), and abnormal breast(s) (bottom).

Compared with tumor segmentation, whole-breast segmentation is a simple task due to the clear intensity contrast. In this paper, we adopt the U-Net structure for whole-breast segmentation, considering its great segmentation accuracy and generalization. As expected, the overall breast segmentation performance is excellent, i.e., achieving a DSC of 92.6% on the internal testing set as well as the external sets. Representative segmentation results are given in Figure 2. For each center, we show three typical cases, including standard-sized breasts, small-sized breasts, and abnormal breast(s) (including only one breast due to surgical removal of the other breast or breast implant surgery). From Figure 2, it is evident that the quality of whole-breast segmentation is robust for different breasts from different centers. A detailed analysis about the benefits of whole-breast segmentation will be given in the ablation study section.

### Breast tumor segmentation performance

Given the DCE-MRI with multiple phases, we use both the original images and the subtraction images (i.e., between the corresponding images with and without contrast agent injection) as the input to capture dynamic spatial-temporal information via a spatial-temporal transformer. Equipped with a large amount of training data and the excellent performance of transformers,^39^ the proposed model could be better generalized to achieve robust segmentation of images acquired by different imaging protocols and carrying varied tumor categories.

**Figure 3:**
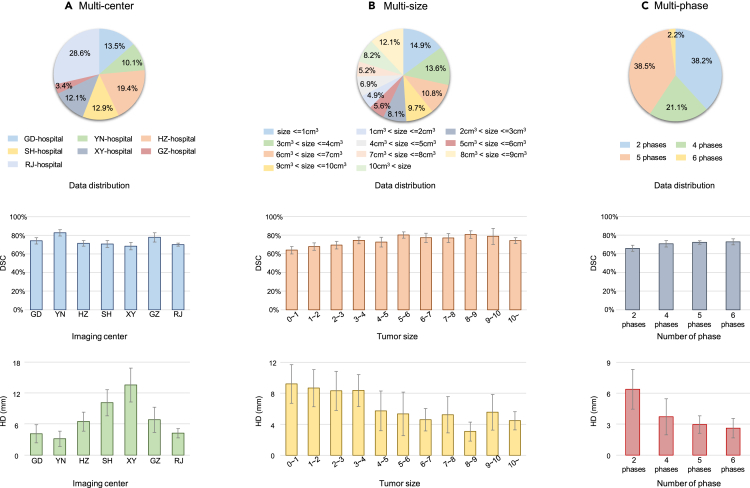
Tumor segmentation performance on the testing sets (A) Segmentation performance on seven different centers. (B) Segmentation performance on different sized tumors. (C) Segmentation performance with respect to different phase number. The pie charts in the first row show the data distribution, and the bar charts in the second and third rows show DSC (%) and HD (mm), respectively. The error bars indicate the 95% confidence interval.

**Figure 4:**
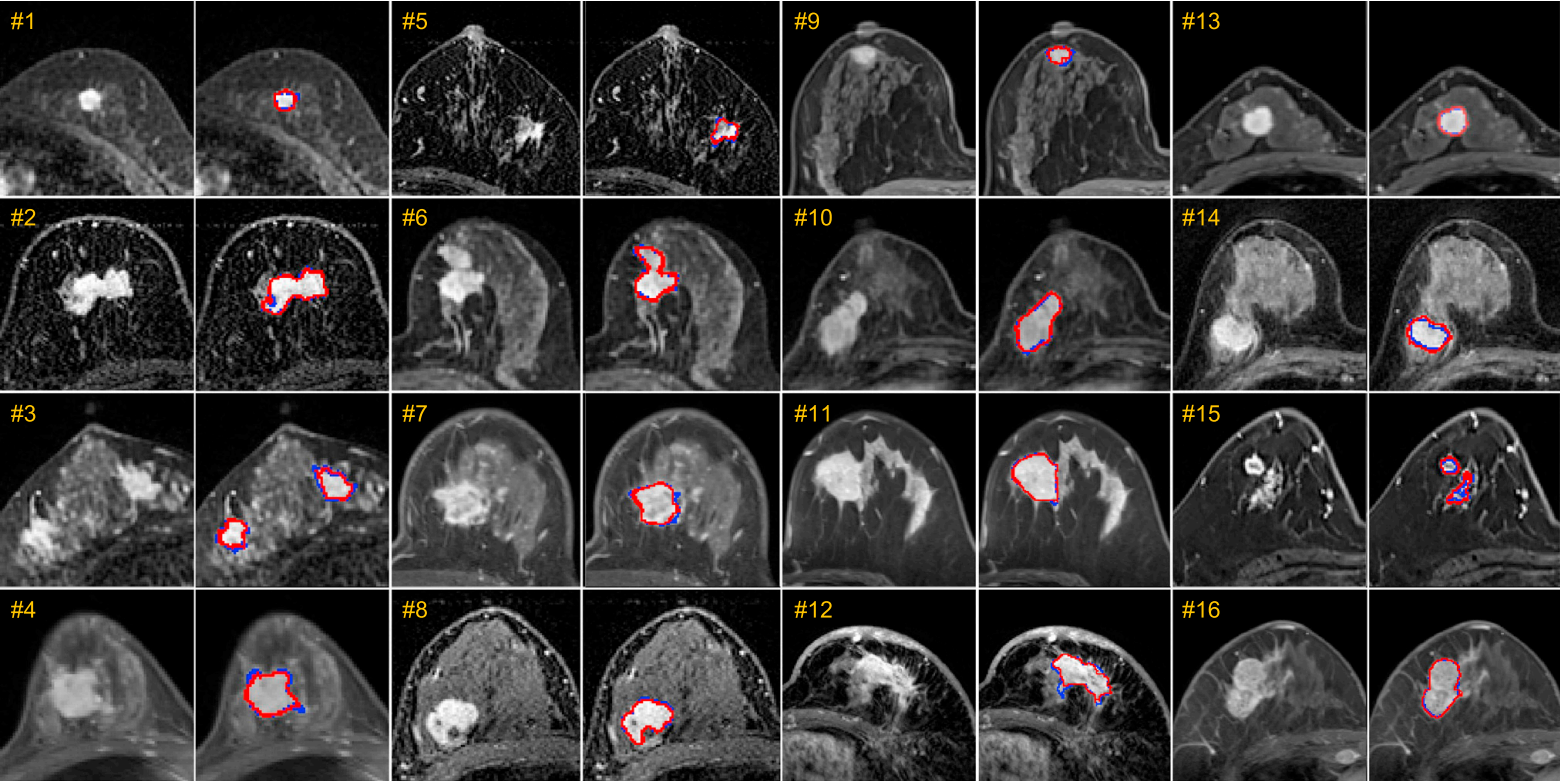
Typical examples of breast tumor segmentation on the testing set Cases 1–8 are from the internal testing set, and cases 9–16 are from the external testing set. For each testing case, we show two images with and without annotations for better comparison. Blue delineation denotes ground truth annotations, while red delineation indicates automated segmentations by our model.

**Figure 5:**
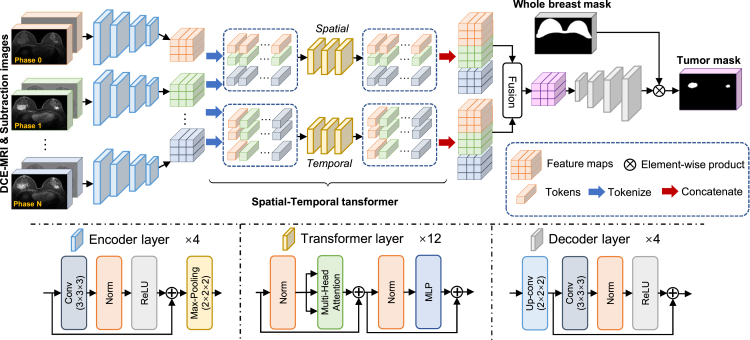
Overview of proposed tumor segmentation models It tasks both original images and subtraction images from all phases as inputs. The weights from encoders in breast tumor segmentation model are shared. The detailed structures of encoder layer, transformer layer, and decoder layer are also illustrated in the figure.

We have evaluated our tumor segmentation model on multi-center testing sets quantitatively and qualitatively. Note these multi-center data are acquired with quite different imaging settings (e.g., with different phase numbers and intervals, voxel sizes, and manufacturers). Quantitative results are provided in Figure 3, and segmentation results are shown in Figure 4, indicating good agreement between the automated segmentations by our model and the manual annotations by radiologists. Particularly, Figure 3A shows the pie chart describing data distribution among different centers (top) along the two bar charts (middle and bottom) describing the DSC and HD for each center. It can be seen that the best performance is achieved on YN-hospital (the internal testing set), with 82.7% and 3.1 mm for DSC and HD, respectively. In contrast, the segmentation results from XY-hospital (external set) are the worst with a DSC of 68.3% and HD of 12.1 mm, respectively. In addition, the segmentation results of all evaluation sets are summarized in Table 2, i.e., with a DSC of 77.7% on the internal testing set and 70.5% on the external testing set and 72.4% on all testing sets. In this table, the performance of our method is also compared with five other methods, while ours achieves the best performance in terms of all metrics. It is noteworthy that although segmentation accuracy decreases on the external set, the DSC of 70.5% is still clinically acceptable for downstream diagnosis and treatment, as confirmed by clinicians.

**Table 2:** Comparison of different methods in breast tumor segmentation on internal and the external testing sets

	Internal testing set			External testing set			All testing sets		
Method	DSC	SEN	HD	DSC	SEN	HD	DSC	SEN	HD
U-Net^40^	72.4 ± 2.6	70.3 ± 2.7	6.1 ± 1.6	59.0 ± 1.7	75.1 ± 1.7	17.2 ± 1.4	62.5 ± 1.4	73.8 ± 1.1	14.3 ± 1.2
MHL^34^	66.5 ± 2.8	62.2 ± 3.0	8.4 ± 2.2	66.5 ± 1.6	67.5 ± 1.9	8.8 ± 1.3	66.1 ± 1.4	66.1 ± 1.6	8.7 ± 1.1
Tumor-sen^36^	70.9 ± 2.7	67.0 ± 2.8	4.8 ± 1.4	59.8 ± 1.7	68.9 ± 1.8	16.0 ± 1.5	62.7 ± 1.4	68.4 ± 1.6	13.1 ± 1.2
ALMN^41^	74.6 ± 2.4	72.5 ± 3.0	6.7 ± 1.2	56.4 ± 1.6	75.0 ± 1.6	19.2 ± 1.3	61.1 ± 1.4	73.7 ± 1.3	15.9 ± 1.1
TSGAN^38^	68.5 ± 1.6	69.4 ± 2.6	8.5 ± 1.5	63.3 ± 1.9	68.4 ± 1.6	19.8 ± 1.0	62.7 ± 1.9	68.7 ± 1.4	12.4 ± 1.2
Ours	77.7 ± 2.4	75.1 ± 2.5	3.7 ± 1.1	70.5 ± 1.3	76.5 ± 1.5	7.6 ± 0.9	72.4 ± 1.1	76.2 ± 1.3	6.6 ± 0.7

The evaluation metrics include DSC (%) ↑ , SEN (%) ↑, and HD (mm) ↓.

We also analyze the robustness of segmentation performance with respect to different tumor sizes. As shown in Figure 3B, we use 1 mm ${}^{3}$ as the interval to divide tumors into 11 categories according to the sizes of the manually labeled tumors by radiologists. The segmentation performance of our AI system is robust for the medium-to-large tumors with 3– 10 mm ${}^{3}$, as well as the large tumors above 10 mm ${}^{3}$, achieving an average DSC of 74.1% and HD of 4.4 mm. For the small-sized tumors, especially those extremely tiny ones (i.e., below 1 mm ${}^{3}$), the overall DSC decreases slightly to 63.9%, and the overall HD increases to 9.20 mm. Note that a tiny segmentation error in small-sized tumors, even with under- or over-segmentation of several voxels, can significantly affect the evaluation metrics. Overall, reasonable segmentation results can be achieved for tumors of various sizes.

We further evaluate the robustness of our AI assistant in handling DCE-MRI data with varied phase numbers. As described above, our AI assistant exploits temporal information across different phases for improved segmentation performance. It is noted that not all cases have six phases available in our dataset (e.g., some cases in the RJ-hospital dataset), due to factors such as image corruption caused by the motions, although these missing-phase cases occur rarely. Fortunately, benefiting from the attention mechanism of the transformer, our framework can flexibly deal with an arbitrary number of image phases by ignoring the attention weights of missing image phases. We summarize the segmentation performance on patients with different phase numbers from RJ-hospital in Figure 3C. Consistent with our expectation, the segmentation performance gets better when more phases are involved. For example, when the phase number increases from two to six, the DSC accuracy is improved from 65.9% to 72.9%, and the HD error decreases from 6.38 to 2.61 mm. These results demonstrate the advantages of our segmentation model in that it (1) is robust to different numbers of image phases, (2) can efficiently exploit temporal relationships across different image phases to improve segmentation performance, and (3) will benefit from more image phases.

To demonstrate the superiority of our model, we also compare our model with other well-established deep-learning-based breast tumor segmentation methods on both the internal testing set and the external testing set, including U-Net,^40^ MHL,^34^ Tumor-sen,^36^ ALMN,^41^ and TSGAN.^38^ Note that the U-Net, MHL, Tumor-sen, and ALMN are 3D tumor segmentation networks that only consider one or two enhancement image(s), ignoring temporal information across different phases. Although TSCAN has attempted to exploit temporal information by designing a graph attention module, the signal dynamics across different phases are still not well explored. Furthermore, it only takes 2D slices as the inputs, so the segmentation consistency across different slices cannot be guaranteed. The quantitative metrics of segmentation results are summarized in Table 2 for each method. It can be seen that our AI assistant achieves the best performance, significantly outperforming other methods, with p values (by pair t tests) lower than 0.05 for all metrics. The performance gain is mainly from our specifically designed spatial-temporal framework to capture dynamic changes across different phases. Furthermore, by leveraging the whole-breast segmentation network, many artifacts (e.g., over-segmentation on non-breast regions) can be avoided, thus contributing to lower HD errors. Overall, our model is robust and generalizable on both internal and external testing sets.

### Ablation study

To segment breast tumors accurately, our AI assistant system contains several key modules, including the whole-breast segmentation model, the spatial transformer, and the temporal transformer. In order to evaluate the contribution of each module, we design an ablation study on all testing sets (Table 3). When adding the whole-breast segmentation model, a significant improvement in DSC and HD metrics is achieved by removing false-positive predictions, especially in non-breast regions. Hence, it is crucial to first localize tumor regions automatically before tumor segmentation. Besides, considering that CNN kernel is a local operation and cannot model long-range relations spatially, we design a spatial transformer in bottleneck to explore long-range spatial dependency within each phase. Moreover, a temporal transformer is also proposed to capture dynamic changes across different phases. Hence, the best performance is achieved by integrating these two transformers to comprehensively exploit spatial-temporal information, leading to 5.1% and 2.7% improvement for DSC and SEN metrics, respectively.

**Table 3:** Evaluation of our specially designed modules in tumor segmentation on all testing sets

	Module			All testing sets		
Model	whole-breast segmentation model	spatial transformer	temporal transformer	DSC	SEN	HD
Baseline				63.1 ± 1.4	72.1 ± 1.2	15.9 ± 1.1
Baseline + breast mask	✓			67.3 ± 1.2	73.5 ± 1.3	7.6 ± 0.8
Baseline + breast mask + S-trans	✓	✓		69.9 ± 1.2	74.2 ± 1.3	6.9 ± 0.9
Baseline + breast mask + T-trans	✓		✓	70.6 ± 1.6	74.2 ± 1.5	7.3 ± 0.8
Baseline + breast mask + ST-trans	✓	✓	✓	72.4 ± 1.1	76.2 ± 1.3	6.6 ± 0.7

The evaluation metrics include DSC (%) ↑ , SEN (%) ↑, and HD (mm) ↓. The modules include whole-breast segmentation model, spatial transformer, and temporal transformer.

### Clinical impact

(1) Tumor segmentation compared with experienced radiologists. The proposed AI assistant for breast tumor segmentation aims to relieve physicians from tedious and cumbersome annotation processes. To demonstrate the segmentation efficiency of our AI assistant, we randomly choose 100 cases from GD-hospital and invite two radiologists (Expert-1 and Expert-2), who do *not* participate in the ground truth labeling process, to annotate breast and tumor regions from scratch and to help refine automatic segmentations by our model, respectively. The segmentation accuracy and efficiency are summarized in Table 4. From this table, first, we can see that our proposed AI assistant can achieve comparable segmentation accuracy to experienced radiologists, but it is more than 20 times faster. This gives radiologists more time to focus on downstream diagnosis and treatment plans. On the other hand, we find significant annotation differences across different radiologists at different times, while our AI assistant can generate more robust segmentations. Meanwhile, manual refinement of automatic annotations from our AI assistant can produce more accurate results (e.g., 5% of DSC improvement from what is achieved by the radiologists or the AI assistant alone) and also significant efficiency improvement over manual annotations from scratch (i.e., seven to 10 times faster). This indicates the advantages of our AI assistant in helping radiologists with fast tumor annotation.

**Table 4:** Comparison of tumor segmentation (in terms of accuracy and efficiency) between our proposed AI assistant and two radiologists

Model	Expert-1	Expert-1^a^	Expert-2	Expert-2^a^	AI assistant
DSC (%)	81.70	86.17	81.15	86.20	82.04
SEN (%)	79.46	82.49	73.82	80.85	75.68
HD (mm)	0.56	0.52	1.69	0.78	1.25
Time (min)	14.28	2.09	15.16	1.42	0.67

a Indicates further manual annotation refinement based on automatic segmentations by our proposed AI assistant. It can be seen that AI + further manual annotation obtains the best result.
(2) Necessity of accurate tumor segmentation for better diagnosis. In clinical practice, physicians usually analyze tumor shapes and positions based on MR images and segmentation masks. For example, the T-stage is an important pathological index to indicate malignancy and prognosis of breast tumors, which is derived from tumor size and position information. Specifically, T-stage has four categories, including (1) T1, i.e., the widest part of the tumor is less than 20 mm; (2) T2, i.e., the widest part of the tumor is more than 20 mm but less than 50 mm; (3) T3, i.e., the widest part of the tumor is more than 50 mm; and (4) T4, i.e., the tumor has spread to the chest wall or skin. During diagnosis, physicians often only check three cross-sectional views to select the widest axis to classify the T-stage. However, this process is inaccurate, since the widest axis does not correspond to the widest part of the actual tumor located in the 3D space. To demonstrate the clinical usefulness of our tumor segmentation mask, we compute the accuracy of T-stage classification on the 100 cases from the radiologists’ classification (1) without segmentation masks, (2) based on the manually annotated segmentation masks, and (3) based on the segmentation masks produced by our AI assistant. Without segmentation masks, the classification accuracies are 81% and 78% by the two radiologists, respectively, which is not unsatisfactory for precise diagnosis. But the accuracy is significantly improved when the tumor masks are involved in T-stage classification. For example, using manually annotated masks, the classification accuracy can be 94% and 93% from the two radiologists, with a comparable accuracy of 94% using the automatic annotations by our AI assistant, indicating the effectiveness of our AI assistant for clinical diagnosis.
(3) AI-based breast cancer diagnosis system. One important research topic in breast cancer is to predict the response after breast cancer neoadjuvant therapy using MRI-based radiomics features, which greatly relies on the tumor annotation from professional radiologists, due to lacking robust segmentation algorithms in the past. To demonstrate the clinical impact of our AI system in reducing the workload of radiologists, we extract radiomics features from manually annotated tumors by experienced radiologists and also automatically annotated tumors by our AI assistant, respectively, to train a classifier to predict neoadjuvant therapy response after standard radiotherapy process.^24^ Specifically, we randomly choose 100 cases from the testing set in YN-hospital, with 34 cases of pathologic complete response (pCR) and 66 cases of non-pCR (npCR). The feature extraction model (https://pyradiomics.readthedocs.io) extracts radiomics features (e.g., shape, histogram, and texture) from DCE-MRI and segmentation masks.^42^ The classifier (i.e., SVM) predicts whether the case is pCR or npCR. From the prediction results with 5-fold cross-validation, we achieve an accuracy of 66.8% with AI annotations, comparable to that of 67.1% with manual annotations. Similar results can be found using the receiver operating characteristic curve (AUC), with 0.79 (AI) versus 0.80 (human), respectively. Hence, we believe our proposed AI assistant can be easily applied to facilitating downstream diagnosis tasks (e.g., molecular subtype prediction^43^^,^^44^^,^^45^ and Ki-67 expression level prediction^46^^,^^47^) to promote the building of an automated all-purpose AI-based breast cancer diagnosis system.

## Discussion

Breast tumor segmentation allows comprehensively characterizing tumor properties (e.g., shape, size, single lesion or multiple lesions) that are crucial for accurate diagnosis and treatment plan. Currently, due to the lack of robust and automatic tumor segmentation tools, most AI-assisted breast cancer diagnosis models (e.g., for predicting breast tumor molecular subtype and response to breast cancer neoadjuvant therapy) still rely heavily on manual intervention of experienced radiologists,^25^^,^^26^^,^^27^^,^^28^ which is time-consuming and tedious.

Though several works have exploited AI-assisted breast tumor segmentation on DCE-MRI data, their clinical availability and robustness are still unproven in 3-fold aspects. First, none of these works release their data, codes, or well-trained segmentation models, hindering their direct application to clinical practice. Second, the segmentation performance of the existing methods is only evaluated on their respective small internal dataset from a single center (e.g., 64 cases for TSGAN, 272 cases for MHL, and 422 cases for ALMN and Tumor-sen). Their robustness and generalizability on a multi-center dataset are unclear. For example, they may encounter over-fitting problems caused by training only on a single-site dataset. Third, most methods only pay attention to the contrast-enhanced tissues in two image phases, ignoring dynamic changes across different phases, although these dynamic changes are crucial to detect some small tumors and distinguish them from other enhanced tissues (e.g., vessels).

Based on these observations, we propose a robust AI assistant for breast tumor segmentation by exploiting signal dynamics across different phases of DCE-MRI data. Specifically, our spatial-temporal framework can well capture both inter-phase and intra-phase long-range dynamic information. Moreover, we also design the whole-breast segmentation model to remove over-segmentations in non-breast regions to boost segmentation performance. Another essential contribution of this work is that we evaluate our AI assistant for breast tumor segmentation on a very large dataset (13,167 MR scans from 2,197 cases) collected from seven medical centers. We would like to emphasize that the data used in this study is pretty large, with various data distributions compared to most existing works. Although the authors in the paper^48^ used a large-scale dataset, they collected data only from a single center without external evaluation. Hence, the advantage of multi-center evaluation in our paper makes our study more reliable. As shown in Table 2 and Figure 3, we have demonstrated impressive robustness of our method in addressing varied imaging protocols (especially different phase numbers) and tumor sizes. For all experiments conducted on this large dataset, our AI assistant achieves the best performance among all competitive segmentation methods. Most importantly, the generalizability of our AI assistant can be validated by its stable performance on the external testing dataset. In addition, we also achieve better or similar results (i.e., with DSC of 77.7%) on the internal testing set, when comparing with the reported performance of the competitive methods, such as 51.6% by random forest,^49^ 63.5% by TSGAN,^38^ 68.8% by SegNet,^50^ 71.7% by MHL,^34^ 75.88% by ST3D-net,^51^ 77.0% by GOCS-DLP,^35^ 78.0% by ALMN,^41^ and 78.7% by Tumor-sen.^36^ The code and the trained models of our AI assistant will be released to promote breast cancer-related research or non-commercial clinical applications. Users can directly test their own data with our trained models or train the model from scratch.

Besides, our proposed tumor segmentation model is also robust to variations in DCE-MRI imaging intervals, i.e., 1 min for most centers and 45 s for RJ-hospital, by capturing dynamic signal changes across different phases with temporal information. In addition, we also demonstrate the model’s robustness when some image phases are unavailable or missing (see Figure 3C for details). The specially designed temporal transformer can deal with an arbitrary number of inputs by ignoring the attention weights of missing phases, benefiting from the flexibility of the attention mechanism originated from natural language processing and widely adopted in computer vision fields.^52^ It is noteworthy that motion during breast DCE-MRI imaging is often relatively small. Thus, registering all phase images brings little accuracy gain but takes much longer computational time, which is thus not adopted in our framework. This somewhat also indicates the robustness of our model to motions during the scanning process.

Finally, we would like to emphasize again that our proposed robust and efficient AI assistant is also beneficial for clinical applications. First, our accurate tumor segmentation mask can help physicians obtain more morphological information about tumors, such as tumor size, location, and malignancy, without tedious manual annotations. Second, automated segmentation is the primary step to build a smart AI diagnosis system for breast cancer. Lacking open-source breast tumor segmentation software makes existing breast tumor diagnosis research rely on manual annotations from radiologists. Our framework aims to relieve radiologists from this manual annotation step and allow their efforts to be devoted to subsequent diagnostic tasks. For example, predicting the response after breast cancer neoadjuvant therapy based on radiomics features extracted from our tumor segmentation masks can achieve comparable performance with that using manual annotations (see more details in the clinical impact section). And we believe this robust segmentation model can be further applied to other tasks, such as predicting molecular subtypes of breast tumors.

In the section evaluating clinical impact, we compared tumor segmentation accuracy with experienced radiologists. We also evaluated two related works^34^^,^^48^ with reportedly similar performance as radiologists. It is worth mentioning the difference between the segmentation evaluation methods used in our work and the two mentioned works. Specifically, two mentioned works re-used annotations from each individual radiologist as partial ground truth to train the network. While, in our work, we invited two new radiologists (who did *not* participate in the annotation process) to label images from scratch; thus our evaluation is more strict.

In conclusion, the proposed AI assistant allows robust and efficient breast tumor segmentation. We believe this model can promote more research and serve as a crucial part of the AI-assisted breast cancer diagnosis system.

### Limitations of study

In this study, our AI assistant was trained on data from Asian populations, which typically have denser breasts with more fibroglandular tissue. In the future, we plan to add more diverse data from other populations to evaluate segmentation performance. Additionally, this study focused solely on segmentation from DCE-MRI, neglecting the important role of other MRI sequences (such as T2 weighted images and diffusion-weighted images) that provide tumor region characteristics from other perspectives. To further improve accuracy, a multi-parametric MRI tumor segmentation system is needed, and we are currently working on its design.

## Methodology

### Data pre-processing

This study was approved by the Research Ethics Committee from all centers. A detailed description of the dataset has been provided in Table 1. Considering large variations of spatial resolutions across different centers and also intensity distributions across different manufacturers, we, respectively, apply spatial normalization and intensity normalization in the data pre-processing step. Specifically, we interpolate all data to the same resolution with a voxel size of 1.0×1.0×1.0 ${\text{mm}}^{3}$. For intensity normalization, we clip the outliers (top 1% of the maximum values) of all images at different phases and perform min-max normalization on phase 2, which typically exhibit the strongest enhancement. The maximum and minimum values from the phase 2 images are then used to normalize the images at other phases, which makes all intensity values range from 0 to 1 while preserving the intensity change information across time sequences. Limited by the GPU memory, we extract many overlapped 3D patches to train the segmentation model. For the whole-breast segmentation model, we randomly crop patches with the size of 128×128×48. For the breast tumor segmentation model, we crop patches with the size of 96×96×48. Besides, since the number of background voxels significantly exceeds the foreground voxels, we require each cropped patch to contain foreground areas according to the training manual tumor annotations for efficient training of the segmentation model.

#### Whole-breast segmentation model

Compared with tumor segmentation, whole-breast segmentation is straightforward. We adopt the U-Net structure for breast segmentation. Patches of pre-contrast DCE-MRI data are fed into the U-Net to get the final breast mask. The detailed structure is the same as the work^40^ with four downsampling layers and upsampling layers, and the skip connections of corresponding layers can exploit latent semantic information with more details. We adopt the DICE loss function^53^ to supervise the model training.

#### Tumor segmentation model

In this paper, we propose to combine both the spatial information within each phase and the temporal information across different phases in DCE-MRI data. An overview of our network is shown in Figure 5. Briefly, we first use a convolution-based encoder E to get global spatial features in each phase, and we further design a spatial-temporal transformer to combine the features across different phases to capture the spatial-temporal information. The combined spatial-temporal features are then sent through a convolution-based decoder D to predict the tumor segmentation mask.

Specifically, we use ${\left\{x^{i}\right\}}_{i=0}^{N}\in R^{\left(N+1\right)\times L\times W\times H}$ to denote multi-phase DCE-MRI data, where $x^{0}$ is the pre-contrast image and the others are the *N* post-contrast images. The symbols L, *W*, and *H* correspond to the length, width, and height of an input patch, respectively. We use $y\in R^{1\times L\times W\times H}$ to denote corresponding manual annotation mask. Considering the importance of subtraction images of DCE-MRI in manual tumor annotation, we also use the subtraction images at each phase as additional inputs, which can be calculated by ${\left\{s^{i}=x^{i}-x^{0}\right\}}_{i=0}^{N}\in R^{\left(N+1\right)\times L\times W\times H}$. We input both original DCE-MRI and the subtraction images into the encoder E to get feature maps for each phase and denote them as ${\left\{f^{i}=E\left(x^{i},s^{i}\right)\right\}}_{i=0}^{N}\in R^{\left(N+1\right)\times C\times L^{\prime }\times W^{\prime }\times H^{\prime }}$. Note that, $C,L^{\prime },W^{\prime },H^{\prime }$ are the channel number, length, width, and height of feature maps, respectively. The feature maps at different phases are then tokenized to form the spatial tokens $z_{spatial}\in R^{\left(N+1\right)\times K\times C}$ and the temporal tokens $z_{temporal}\in R^{K\times \left(N+1\right)\times C}$, where $K=L^{\prime }\times W^{\prime }\times H^{\prime }$. For the spatial token $z_{spatial}$, we treat the first dimension (N+1) as the size of the mini-batch, the second dimension *K* as the number of tokens, and the last dimension *C* as the length of each token. In this way, we could well represent spatial information. Differently, the temporal token $z_{temporal}$ is composed of *K* batches, with each batch containing N+1 tokens, and each token having a length of *C*, which can comprehensively represent dynamic temporal information across different phases. The further proposed spatial-temporal transformers ($T_{s}$ and $T_{t}$) can exploit the latent relationship among spatial tokens and temporal tokens to improve segmentation performance via the attention mechanism. Specifically, the output tokens can be expressed as ${\tilde{z}}_{spatial}=T_{s}\left(z_{spatial}\right)\in R^{\left(N+1\right)\times K\times C}$ and ${\tilde{z}}_{temporal}=T_{t}\left(z_{temporal}\right)\in R^{K\times \left(N+1\right)\times C}$. We restore the feature maps $\tilde{f}\in R^{C^{\prime }\times L^{\prime }\times W^{\prime }\times H^{\prime }}$ by combining spatial-temporal tokens together and use convolution layers to reduce phase dimension, where $C^{\prime }$ is the channel number of new feature maps. Then, we can get segmentation results ($\tilde{y}\in R^{1\times L\times W\times H}$) from the decoder D, which is $\tilde{y}=D\left(\tilde{f}\right)$. In order to remove the over-segmentation in the non-breast regions, the final tumor segmentation results are masked by the whole-breast segmentation region for effective removal of false-positive segmentations.

In our segmentation model (Figure 5), we choose the U-Net structure with residual blocks for the encoder E and the decoder D. Four downsampling and upsampling blocks are adopted. For the spatial and temporal transformers, we use the same architecture as the vision transformer,^39^ and the number of transformer layers is 12. We use both DICE and binary cross-entropy loss functions to supervise the training of tumor segmentation model.

#### Training details

All experiments are conducted on the PyTorch platform with two NVIDIA TITAN RTX GPUs (24GB). We use the ADAM optimizer to optimize all networks. The initial learning rates of the whole-breast and tumor segmentation models are 0.002 and 0.001, respectively. And, the learning rate decays by half for every 50 epochs. A total number of 300 epochs are set for each task. We compute the training loss within 10 epochs to determine the convergence. While using the well-trained segmentation models to test the results, we use sliding windows to crop the overlapping patches, whose stride is half of the patch size. Then, we average the overlapping patches to obtain the final results.

## Experimental procedures

### Resource availability

#### Lead contact

Further information and requests for data should be directed to and will be fulfilled by the lead contact, Dinggang Shen (dgshen@shanghaitech.edu.cn).

#### Materials availability

This study did not generate new unique materials.

#### Ethical approval

The data were collected from seven medical centers, including Guangdong Provincial People’s Hospital, Yunnan Cancer Hospital, Hangzhou First People’s Hospital, Shanghai General Hospital, The Second Xiangya Hospital, Guizhou Provincial People’s Hospital, and Ruijin Hospital. This study was approval by the ethical committee of each medical center. Due to the retrospective nature of this study, the informed consent was waived by the relevant institutional review board.

## Data Availability

The full datasets are protected because of privacy issues and regulation policies in hospitals. Partial data can be accessible to support the results in this study, with permission from respective medical centers. The released data can be download via Zenodo^54^ (https://doi.org/10.5281/zenodo.8068383). The codes and inference version of the breast AI assistant are also accessible via Zenodo^54^ (https://doi.org/10.5281/zenodo.8059654).
